# Case Report: A case of type II cryoglobulinemia secondary to diffuse large B-cell lymphoma

**DOI:** 10.3389/fonc.2026.1796461

**Published:** 2026-04-22

**Authors:** Cuiping Mao, Xiaohe Hao

**Affiliations:** Department of Clinical Laboratory, Shandong Cancer Hospital and Institute, Shandong First Medical University and Shandong Academy of Medical Sciences, Jinan, Shandong, China

**Keywords:** cryoglobulinemia, diffuse large B-cell lymphoma, non-HCV infection, pseudo-elevation of platelets, zuberitamab

## Abstract

**Background:**

Diffuse Large B-Cell Lymphoma (DLBCL) represents the most prevalent subtype of Non-Hodgkin Lymphoma (NHL). Cryoglobulinemia results from reversible precipitation of serum immunoglobulin complexes at low temperatures, with Type II (mixed) cryoglobulinemia commonly associated with hepatitis C virus (HCV) infection, autoimmune diseases, and B-cell lymphoproliferative disorders. However, its occurrence secondary to DLBCL is exceedingly rare. This report presents a rare case of DLBCL complicated by Type II cryoglobulinemia, acute liver injury, acute kidney injury, and severe infection.

**Case report:**

A female patient in her 40s presented to an external hospital with unexplained high fever, dry cough, and chest tightness. She was diagnosed with DLBCL based on bone marrow cytology and histopathological findings. The patient received chemotherapy with the zuberitamab plus CHOP regimen. However, anaphylactic shock occurred during zuberitamab infusion, leading to temporary discontinuation of chemotherapy. Upon transfer to our hospital, laboratory tests indicated renal insufficiency and severe hepatic injury. Combined with clinical manifestations such as extensive skin and mucosal ecchymoses, bilateral lower limb edema, arthralgia, reversible serum cryoprecipitation, positive mixed type II cryoglobulin, and decreased C4 complement levels, a diagnosis of type II cryoglobulinemia was confirmed. Following fluid resuscitation, diuresis, and continuous renal replacement therapy, her condition temporarily improved. Unfortunately, the patient succumbed due to disease progression of lymphoma.

**Conclusion:**

This case illustrates the diagnostic and therapeutic management of type II cryoglobulinemia secondary to DLBCL. It highlights the importance of closely monitoring clinical manifestations and implementing early intervention for related complications during the treatment of B-cell lymphoproliferative disorders, aiming to reduce the high mortality rate and improve patient outcomes.

## Introduction

Cryoglobulins are immunoglobulin complexes that precipitate spontaneously at temperatures below 37 °C and redissolve upon warming above 37 °C. Their composition may include monoclonal immunoglobulins, polyclonal immunoglobulins, rheumatoid factors, among others. The presence of cryoglobulins in the blood is referred to as cryoglobulinemia. Based on the immunoglobulin composition, cryoglobulinemia is classified into three types: I, II, and III. Type I, the monoclonal type, typically consists of monoclonal IgM or IgG and is often associated with lymphoproliferative disorders. Type II, the mixed monoclonal-polyclonal type, frequently comprises IgMκ, IgGκ, and IgGλ, and is commonly linked to systemic autoimmune diseases, lymphoproliferative disorders, and chronic infections. Type III, the mixed polyclonal type, shares similar etiological factors with Type II (1, 2). Cryoglobulinemia often occurs secondary to various underlying diseases, with hepatitis C virus (HCV) infection being the most common and significant etiology (3–5). Additionally, it may be associated with other infectious diseases, systemic autoimmune disorders (such as rheumatoid arthritis, Sjögren’s syndrome, and systemic lupus erythematosus), and B-cell lymphoproliferative diseases (1, 6). In some patients with no identifiable cause, there may be an underlying indolent B-cell lymphoproliferative disorder requiring long-term follow-up due to the risk of transformation into lymphoma (2, 7).

Mixed cryoglobulinemia (Types II and III) is most frequently secondary to HCV infection and is also closely associated with hematologic malignancies, particularly low-grade B-cell lymphoproliferative disorders (2, 7). Studies indicate that approximately 80%–90% of mixed cryoglobulinemia cases are related to chronic HCV infection (8–10). The remaining 10%-20% of non-HCV-related cases are primarily attributed to B-cell malignancies, other infectious diseases, and autoimmune conditions (11).

Diffuse large B-cell lymphoma (DLBCL) is an aggressive non-Hodgkin lymphoma (NHL) derived from mature B-cells, accounting for 35%-40% of all NHL cases and exhibiting significant clinical and biological heterogeneity (12, 13). Existing research indicates a strong association between cryoglobulinemia and B-cell lymphoproliferative disorders. However, most reported cases are in the context of HCV infection (3–5), while instances of cryoglobulinemia secondary to DLBCL are rare, particularly in non-HCV-related settings (14). Currently, there is a lack of systematic understanding regarding the clinical features, diagnostic challenges, and treatment strategies for cryoglobulinemia secondary to DLBCL. This article presents a rare case of Type II cryoglobulinemia secondary to DLBCL. Through a review of the literature and a detailed analysis of the patient’s diagnostic and therapeutic course, we aim to summarize key diagnostic considerations and treatment experiences to provide clinical insights for managing similar cases.

## Case presentation

**Figure 1:**
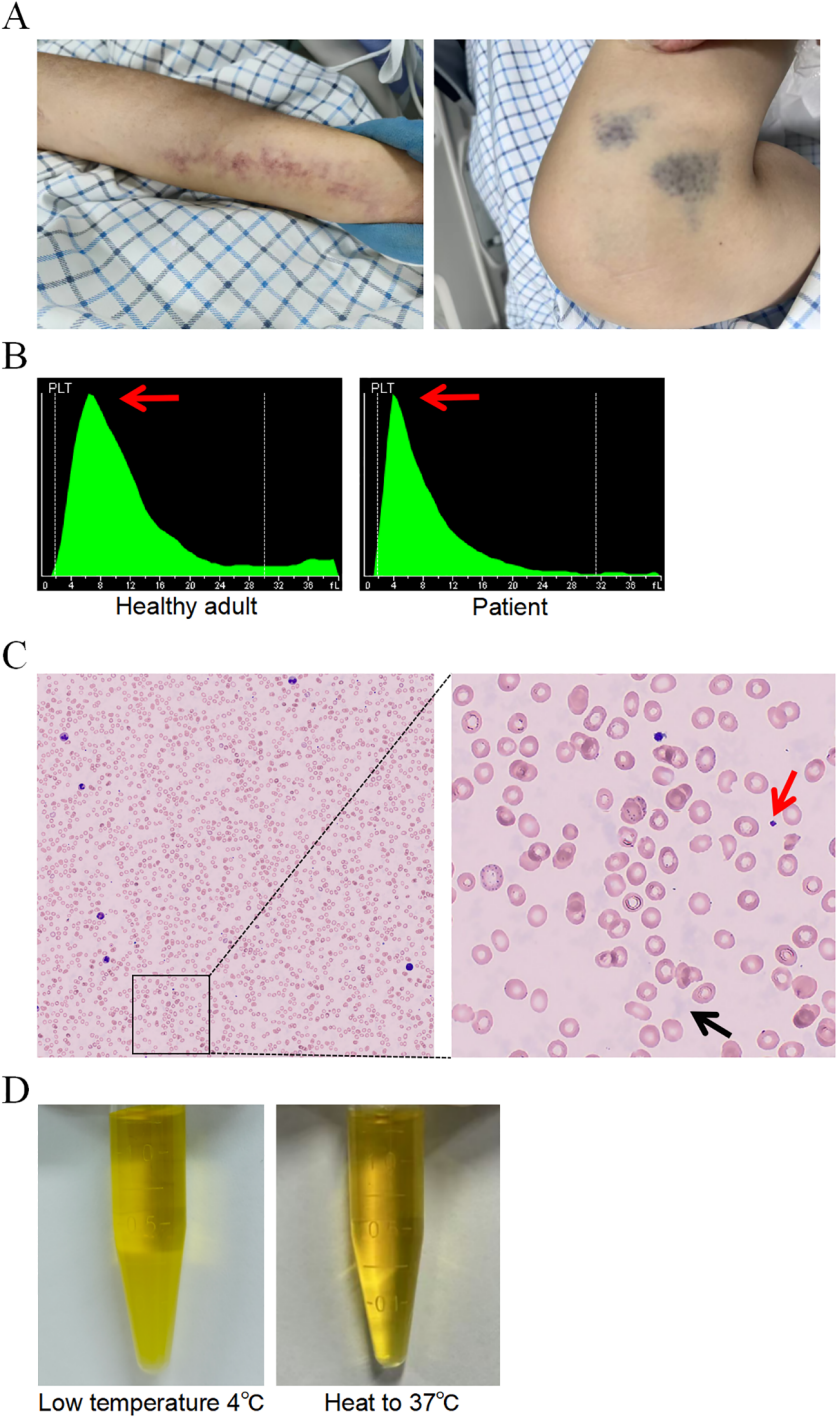
**(A)** Evident purpura was observed on the patient’s upper and lower limbs. **(B)** Platelet histogram from a healthy adult (left) and from a patient with cryoglobulinemia. **(C)** Complete blood count microscopy image. In the right figure, the red arrow indicates platelets, and the black arrow indicates cryoglobulin. **(D)** Patient’s serum at low temperature 4 °C (left) and heated to 37 °C (right).

The patient is a female in her 40s who presented to a primary hospital with fever (peak temperature 38 °C), severe back pain, dry cough, and intermittent chest tightness without any identifiable triggers. Initial complete blood count (CBC) showed a white blood cell count of 4.7×10^9^/L, hemoglobin of 84 g/L, and platelet count of 62×10^9^/L. Physical examination revealed palpable lymphadenopathy in the right axilla, characterized by firm consistency and poor mobility. Computed tomography (CT) of the upper abdomen showed multiple enlarged lymph nodes, splenomegaly, cholestasis, and gallbladder wall edema. Results from bone marrow cytology, flow cytometry, and Histopathologic examination of the bone marrow examinations all indicated invasion of the bone marrow by B-cell lymphoma. To further clarify the nature of the lymph nodes, a right axillary lymph node aspiration biopsy was performed, and the pathological diagnosis confirmed diffuse large B-cell lymphoma (DLBCL). Additionally, electrocardiogram (ECG) findings revealed sinus tachycardia. The patient reported receiving treatment with the combination regimen of zuberitamab plus CHOP at the primary hospital (a local tertiary general hospital). However, an anaphylactic shock occurred during the infusion of zuberitamab, leading to an incomplete course of chemotherapy. Upon admission to our hospital, the patient presented with anemic facies, scattered petechiae and patchy ecchymoses on the skin and mucous membranes, coarse breath sounds with audible moist rales in both lungs, and edema of both lower limbs. Repeated blood tests showed a white blood cell count of 1.24×10^9^/L, hemoglobin of 49 g/L, and a platelet count reported as 726×10^9^/L (**Figure 1A**). However, the platelet histogram displayed an abnormally sharp peak (**Figure 1B**). After manual microscopic review and verification using a Sysmex analyzer, the actual platelet count was determined to be 42×10^9^/L, and the sample was noted to contain flocculent protein interference (**Figure 1C**). Further investigation revealed the presence of reversible cryoprecipitation in the serum (**Figure 1D**). The erythrocyte peak pattern appeared normal. Bone marrow aspiration and biopsy indicated erythroid hyperplasia with normal morphology, and the mature red blood cells exhibited a normocytic, normochromic appearance. Moreover, laboratory tests showed alterations in liver and kidney function parameters as detailed in **Table 1**: Blood urea nitrogen (BUN) was 37.3 mmol/L, Serum creatinine was 169.8 μmol/L, and urine sediment microscopy revealed a red blood cell count >100 per high-power field (HPF), suggesting significant renal impairment. Cardiac troponin T (TnT) was elevated at 16.51 pg/mL (reference range: 0-14 pg/mL), N-terminal B-type natriuretic peptide (NT-BNP) was markedly elevated at 7216 pg/mL (reference range: 0-125 pg/mL), Lactate dehydrogenase (LDH) was elevated at 985 U/L (reference range: 109-245 U/L), and α-hydroxybutyrate dehydrogenase (HBDH) was elevated at 821 U/L (reference range: 72-182 U/L), all indicating myocardial injury. Concurrently, procalcitonin (PCT) was elevated at 8.27 ng/mL (reference range: 0-0.05 ng/mL), and C-reactive protein (CRP) was elevated at 28.39 mg/L (reference range: 0-10 mg/L), suggesting a possible secondary infection or inflammatory state. Virology tests for HBV, HCV, and HIV were all negative.

**Table 1:** Clinical chemistry and immunology tests.

Biomarker	On admission	After treatment	Reference range
Liver function indicators			
ALT	88.4	39.7	7-40U/L
AST	52.8	30.9	15-35U/L
TBIL	22.5	25.3	3.4-17.1μmol/L
DBIL	16.2	15.5	0-3.4 μmol/L
Kidney function indicators			
UREA	37.3	21.2	2.9-8.2mmol/L
CREA	169.8	67.3	41-73μmol/L
UA	778.2	233.5	155-357μmol/L
β2-MG	18.63	6.94	1.3-2.7mg/L
Myocardial function indicators			
LDH	985	345	109-245U/L
HBDH	821	261	72-182U/L
TNT	16.51	14.78	0-14pg/ml
NT-proBNP	7216	5232	0-125pg/ml
Infection indicators			
PCT	8.27	0.29	0-0.05ng/ml
CRP	28.39	10.22	0-10mg/L

**Figure 2:**
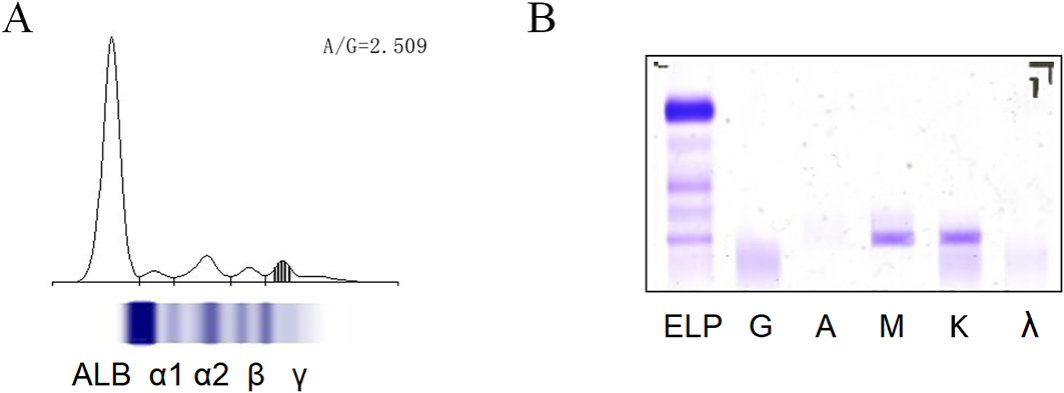
**(A)** Serum protein electrophoresis profile. **(B)** Serum Immunofixation Electrophoresis Profile.

To establish the diagnosis of cryoglobulinemia, immunofixation electrophoresis was performed. Results were negative for Immunoglobulin A (IgA), Immunoglobulin G (IgG), and Immunoglobulin M (IgM), but showed significantly elevated α1-globulin and M-protein levels, with positive findings for IgM-type M-protein and Kappa light-chain M-protein (**Figures 2A, B**; **Table 2**). Complement C3 and C4 levels were significantly reduced (C3: 0.24 g/L, C4: <0.06 g/L), and rheumatoid factor (RF: 26.51 kU/L) activity was elevated (**Table 2**). Furthermore, both lupus anticoagulant and antinuclear antibody results were negative (**Table 3**). Based on the integration of clinical manifestations and laboratory findings, the patient was diagnosed with Type II cryoglobulinemia.

**Table 2:** Autoimmune-related antibody testing.

Biomarker	On admission	Reference range
IgG	5.20	8.6-17.4g/L
IgA	0.57	1.0-4.2g/L
IgM	2.69	0.5-2.8g/L
α1globulin	1.78g/L	/
Percentage of α1globulin	3.8%	1.0-3.2
M protein content	1.9g/L	/
Percentage of M protein	4.0%	/
C3	0.24	0.7-1.4g/L
C4	<0.06	0.1-0.4g/L
RF(rheumatoid factors)	26.51	≤20.0kU/L

**Table 3:** Expression of lupus anticoagulants.

Biomarker	On admission	Reference range
Lupus anticoagulant screening ratio(dRVVT)	1.23	NA
Lupus anticoagulant confirmatory Ratio(dRVVT)	1.35	NA
Lupus Anticoagulant Standardized Ratio(dRVVT)	0.91	<1.2
Lupus anticoagulant screening ratio(SCT)	1.22	NA
Lupus anticoagulant confirmatory Ratio(SCT)	1.26	NA
Lupus Anticoagulant Standardized Ratio(SCT)	0.97	<1.16
ANA	–	–
AHA	–	–
Anti-Sm	–	–
ACA-IgA	<2.50	<8.00APLU/mL
ACA-IgG	<5.00	<8.00GPLU/mL
ACA-IgM	<5.00	<8.00MPLU/mL

Imaging studies further revealed generalized lymphadenopathy (maximum short-axis diameter approximately 3.2 cm), splenomegaly, bilateral pulmonary inflammation, and minimal pleural effusions with ascites in the abdominal and pelvic cavities. Histopathologic examination of the bone marrow findings corroborated the diagnosis of B-cell non-Hodgkin lymphoma.

Upon admission, initial management included symptomatic support therapy such as hepatoprotection, urinary alkalization, and hydration, alongside antimicrobial treatment. However, clinical symptoms showed no improvement, with progressive deterioration in renal and cardiac function. Aggressive diuretic therapy yielded suboptimal outcomes. Subsequently, the patient exhibited worsening generalized ecchymoses. Considering the history of type II cryoglobulinemia and anemia, the Clinical Decision Support System (CDSS) score was assessed as 9 points, leading to transfer to the Intensive Care Unit (ICU). During ICU stay, fluid resuscitation, diuresis, and continuous renal replacement therapy were administered, complemented by intensified anti-infective regimens, hematopoietic stimulation, and transfusion support. The condition temporarily stabilized. Nonetheless, due to poor overall health status and lack of effective antitumor therapy for diffuse large B-cell lymphoma-only conservative management was maintained-the patient developed exacerbated dyspnea with progressively declining heart rate and blood pressure, indicative of acute respiratory failure. Despite aggressive resuscitation, symptomatic improvement was limited. The patient ultimately succumbed during ICU care due to critical illness.

## Discussion

The occurrence of type II cryoglobulinemia secondary to DLBCL is exceedingly rare, with particularly few documented cases in non-HCV-infected individuals. The patient in this case presented with multisystem involvement, including cutaneous purpura, bilateral lower-limb edema, renal insufficiency, and severe hepatic impairment. Pathological and laboratory findings confirmed a diagnosis of type II cryoglobulinemia secondary to DLBCL. During treatment with the zuberitamab plus CHOP regimen, the patient developed a severe allergic reaction. Despite active interventions such as fluid resuscitation, diuresis, and hemofiltration, the patient unfortunately succumbed due to disease progression. A review of this case aims to highlight the diagnostic challenges and clinical implications of cryoglobulinemia associated with aggressive lymphoma, offering valuable insights for the diagnosis and management of type II cryoglobulinemia secondary to DLBCL.

The presence of cryoglobulins may artifactually elevate platelet counts, whereas platelets play a critical role in thrombosis and hemostasis (15, 16). Rapid diagnosis of cryoglobulinemia and accurate determination of platelet levels are therefore essential for timely clinical intervention. In this case, a major confounding factor during initial evaluation was the discordance in platelet counts-pseudothrombocytosis induced by cryoglobulin interference (instrument-reported value: 726×10^9^/L; manual recount: 42×10^9^/L)-while the patient exhibited clinical signs of ecchymosis and bleeding tendency, indicating potential assay interference. Undetected, this discrepancy could lead to misguided clinical decisions. This case underscores the importance of suspecting cryoglobulin interference and performing manual verification when laboratory results are inconsistent with clinical manifestations.

In general, the management of cryoglobulinemia should prioritize addressing the underlying disease and providing symptomatic supportive care, with concomitant advice for patients to avoid cold exposure. For patients with HCV-associated cryoglobulinemia, direct-acting antiviral agents (DAAs) should be selected as etiological therapy (7, 17–19). Regarding symptomatic management, nonsteroidal anti-inflammatory drugs (NSAIDs) constitute the first-line treatment for arthralgia, while corticosteroids (e.g., oral prednisone) can be administered for conditions such as purpura and arthritis. In severe or refractory cases, immunosuppressants may be used in combination (7, 19). For patients with coexisting potential lymphoproliferative or autoimmune disorders, targeted therapy specific to the primary disease-including chemotherapy, targeted agents, and immunomodulators-should be implemented (19–24). Plasmapheresis can serve as an adjuvant intervention and should be combined with an anti-CD20 monoclonal antibody (e.g., Rituximab) to eliminate B-cell clones producing cryoglobulins (25–27). Following a confirmed diagnosis of diffuse large B-cell lymphoma (DLBCL)-induced type II cryoglobulinemia in this case, the patient received chemotherapy consisting of the zuberitamab plus CHOP regimen along with supportive care including fluid replacement, diuresis, and continuous hemofiltration, which initially led to controlled cryoglobulinemia-related damage. However, due to the aggressive progression of the patient’s lymphoma itself and failure to complete systemic anticancer therapy, a fatal outcome ensued. This case underscores that for cryoglobulinemia secondary to lymphoma, systemic treatment directed at the primary lymphoma is fundamental to controlling and even reversing the associated pathological manifestations. Should the primary disease become uncontrolled, the overall prognosis remains poor despite temporary effectiveness of treatments targeting complications.

Therefore, etiological differentiation constitutes a critical step in the diagnosis and management of cryoglobulinemia. Research indicates that mixed cryoglobulinemia is predominantly associated with HCV infection, while non-HCV-related cases are exceedingly rare. Approximately 22% of cases are linked to B-cell lymphoma, with lymphoplasmacytic lymphoma being the most common subtype. In recent years, few cases secondary to DLBCL have been reported (14, 28). Distinct from previous reports, the present case is characterized by an aggressive DLBCL as the primary lymphoma rather than an indolent subtype, along with negative serology for HCV, HBV, HIV and autoimmune antibodies. Furthermore, anaphylactic shock occurred upon the first infusion of the anti-CD20 monoclonal antibody (zuberitamab), leading to interruption of targeted therapy. Additionally, cryoglobulin interference resulted in falsely elevated platelet counts, creating a diagnostic pitfall.

This case has certain limitations. Due to the patient’s critical condition and hemodynamic instability, skin or renal biopsy to confirm cryoglobulinemic vasculitis was not performed. Moreover, following the anaphylactic reaction to the initial zuberitamab infusion and considering the severe thrombocytopenia and active bleeding risk, the standard treatment regimen combining zuberitamab with plasmapheresis could not be attempted. The patient’s short survival period precluded further investigation into the underlying etiology. Nevertheless, these limitations underscore an important avenue for future research: systematic collection and analysis of more non-HCV-associated cryoglobulinemia cases, particularly those linked to aggressive lymphomas such as DLBCL. Notably, prior high-throughput sequencing studies have not identified a specific genetic signature for type II cryoglobulinemia in DLBCL, suggesting that epigenetic dysregulation may play a key role (29). Hence, future studies should focus on epigenetic mechanisms, integrating molecular pathology techniques to elucidate the clonal evolution of B-cell populations driving cryoglobulin production. Such efforts may yield molecular biomarkers for early diagnosis of DLBCL-associated type II cryoglobulinemia, provide a theoretical foundation for targeted therapy, and ultimately improve clinical outcomes in these patients.

## Conclusion

This article presents a rare case of secondary type II cryoglobulinemia in a patient with DLBCL, highlighting the interference of cryoglobulins with platelet detection and the diagnostic challenges associated with multi-organ involvement. Systematic treatment targeting the primary lymphoma is essential for managing this complication, with a particularly poor prognosis when standard therapeutic interventions are hindered.

## Data Availability

The original contributions presented in the study are included in the article/supplementary material. Further inquiries can be directed to the corresponding author.
